# Supplementary material for: Evidence supported by Mendelian randomization: impact on inflammatory factors in knee osteoarthritis
Source: Front Med (Lausanne). 2024 May 28;11:1382836. doi: 10.3389/fmed.2024.1382836 (PMC11165061; doi:10.3389/fmed.2024.1382836)
Supplement: Supplementary file 1 [file Data_Sheet_1.ZIP › data/GCST90274817_5e6/817.leaveoneout.pdf]

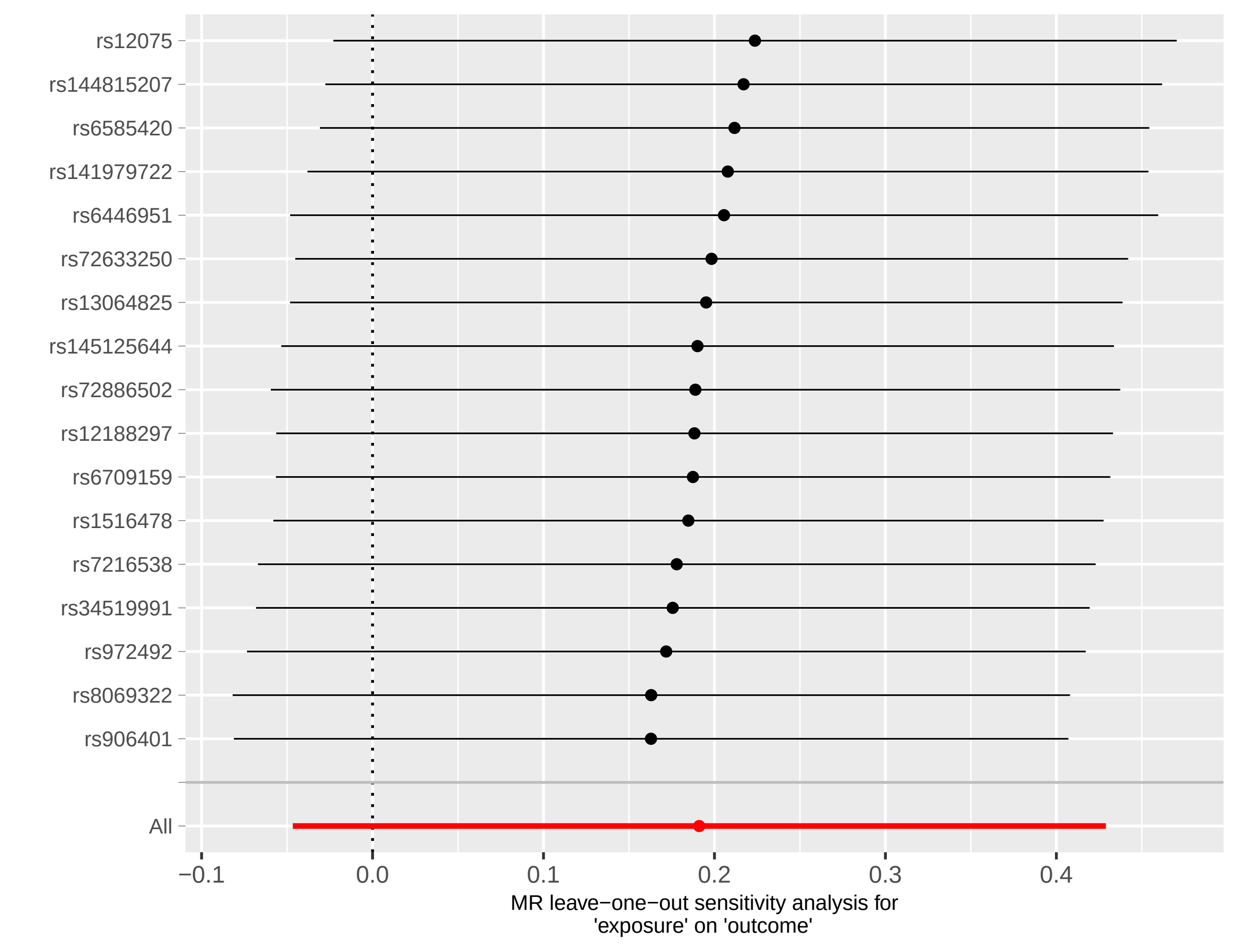

rs12075
rs144815207
rs6585420
rs141979722
rs6446951
rs72633250
rs13064825
rs145125644
rs72886502
rs12188297
rs6709159
rs1516478
rs7216538
rs34519991
rs972492
rs8069322
rs906401
All
−0.1
0.0
0.1
0.2
0.3
0.4
MR leave−one−out sensitivity analysis for
'exposure' on 'outcome'
